# Inhibition of pre-ischeamic conditioning in the mouse caudate brain slice by NMDA- or adenosine A_1_ receptor antagonists

**DOI:** 10.1016/j.ejphar.2012.10.021

**Published:** 2013-01-05

**Authors:** Nikky K. Chauhan, Andrew M.J. Young, Claire L. Gibson, Colin Davidson

**Affiliations:** a School of Psychology, University of Leicester, Lancaster Road, Leicester LE1 9HN, UK; b Division of Biomedical Science, St George's, University of London SW17 0RE, UK

**Keywords:** Dopamine, Voltammetry, MK-801, DPCPX, TTC, HPLC

## Abstract

Evidence suggests that pre-ischeamic conditioning (PIC) offers protection against a subsequent ischeamic event. Although some brain areas such as the hippocampus have received much attention, the receptor mechanisms of PIC in other brain regions are unknown. We have previously shown that 10 min oxygen and glucose deprivation (OGD) evokes tolerance to a second OGD event in the caudate. Here we further examine the effect of length of conditioning event on the second OGD event. Caudate mouse brain slices were superfused with artificial cerebro-spinal fluid (aCSF) bubbled with 95%O_2_/5%CO_2_. OGD was achieved by reducing the aCSF glucose concentration and by bubbling with 95%N_2_/5%CO_2_. After approximately 5 min OGD a large dopamine efflux was observed, presumably caused by anoxic depolarisation. On applying a second OGD event, 60 min later, dopamine efflux was delayed and reduced. We first examined the effect of varying the length of the conditioning event from 5 to 40 min and found tolerance to PIC increased with increasing duration of conditioning. We then examined the receptor mechanism(s) underlying PIC. We found that pre-incubation with either MK-801 or 8-cyclopentyl-1,3-dipropylxanthine (DPCPX) reduced tolerance to the second OGD event. These data suggest that either *N*-methyl-d-aspartate (NMDA) or adenosine A_1_ receptor activation evokes PIC in the mouse caudate.

## 1 Introduction

Around 80% of strokes are ischeamic rather than haemorrhagic (Donnan et al., 2008) in that they involve an interruption of the blood supply to a given region of the brain. Ischeamic stroke causes the death of brain tissue at the site of blood flow interruption and toxicity spreads to surrounding brain tissue as pathological mechanisms, such as glutamate excitotoxicity, progress. At present, treatments for stroke focus on inhibiting or reducing blood vessel occlusions. There are no proven treatments aimed at reducing neurotoxicity per se, despite its potential for inhibiting the consequences of an ischeamic event.

The caudate contains a high level of dopaminergic and glutamatergic input (Graybiel, 1990) and is particularly vulnerable to ischeamic damage. This has been shown both experimentally following middle cerebral artery occlusions in rats (Dijkhuizen et al., 1998; Kumral et al., 1999) and clinically in humans (Caplan et al., 1990; Payabvash et al., 2011). Most of the pre-clinical research on the mechanisms of ischeamic damage has focused on hippocampal or cortical glutamatergic mechanisms, particularly focusing on the glutamate *N*-methyl-d-aspartate (NMDA) receptor. However, in other brain areas, other neurotransmitters, including dopamine, contribute to ischeamic damage. Massive release of dopamine occurs in the caudate in response to anoxia (Kondoh et al., 1995) coinciding with anoxic depolarisation (Toner and Stamford, 1997a) and dopamine levels during ischaemia reach neurotoxic levels (Lieb et al., 1995). Oxygen and glucose deprivation (OGD) evoked dopamine release is partly a consequence of calcium-mediated cell depolarisation via voltage-gated calcium channels resulting from NMDA receptor activation (Toner and Stamford, 1997b; Yoshimoto et al., 2009) as well as reversal of dopamine transporters (Kim et al., 1995) and ATP shortage (Toner and Stamford, 1999). This aberrant release of dopamine from ischeamic neurons exerts neurotoxic effects through the generation of free radicals (Lancelot et al., 1995). Thus, like glutamate in the hippocampus, not only is caudate dopamine release a consequence of ischaemia, it can also cause further neurotoxicity.

Pre-ischeamic conditioning (PIC) is an endogenous neuroprotective mechanism by which a sub-lethal conditioning event provides tolerance to a subsequent ischeamic event (Dirnagl et al., 2009). We have previously examined OGD-evoked dopamine efflux in mouse (Davidson et al., 2011a) and rat (Davidson et al., 2011b) caudate brain slices and showed tolerance to OGD in the caudate 60 min after a 10 min OGD PIC event (Davidson et al., 2011b). This is an important finding as few studies have examined PIC in the caudate, but focus mostly on the hippocampus and cortex. Although the exact mechanisms underlying PIC in the brain are unknown, glutamate is most likely a primary mediator. It is a key neurotoxic transmitter in the sequence of stroke pathophysiology acting on α-amino-3-hydroxy-5-methylisoxazole-4-propionic acid (AMPA) and NMDA receptors to induce massive calcium influx. However, sub-lethal concentrations of NMDA may provide neuroprotection against a further and more severe excitotoxic event (Jiang et al., 2003), and NMDA receptor blockade attenuates these effects in cortical cell culture (Grabb and Choi, 1999). Both adenosine A_1_ receptors and NMDA-type glutamate receptors have been shown to be involved in PIC in vitro (Perez-Pinzon et al., 1996; Kasischke et al., 1996) and in vivo (Saleh et al., 2009; Nakamura et al., 2002), and may act through ATP-dependent K^+^-channels (Perez-Pinzon and Born, 1999), to bring about down-regulation of neuronal metabolic activity, and through Ca^2+^-dependent nitric oxide signalling (Centeno et al., 1999), reducing neuronal susceptibility to death. Understanding the neurochemical mechanisms underlying PIC's protective effects may suggest novel pharmacological strategies for neuroprotection in vulnerable individuals.

The present study used fast cyclic voltammetry in mouse caudate slices to further examine the effect of PIC on OGD-evoked dopamine release. Dopamine efflux after OGD in rodent caudate slices has been shown to be very similar to that seen in vivo after cardiac arrest (Toner and Stamford, 1996). We aimed to (1) ascertain the optimum PIC duration at which neuroprotection to subsequent OGD insult is conferred in the caudate; (2) assess whether blockade of adenosine A_1_ or glutamate NMDA receptor signalling affects PIC.

## 2 Materials and methods

### 2.1 Animals

Adult male mice (C57BL/6; c30 g) kept on a 12/12 h light dark cycle with freely available food and water were killed by cervical dislocation with no anaesthesia, an approved schedule 1 method. Anaesthetics are neuroprotective in this model (Mathews et al., 2001, Toner et al., 2001).

### 2.2 Brain slice preparation

Mouse caudate slices were prepared as previously described (Davidson et al., 2011a). Brains were extracted while irrigated with ice-cold aCSF. A block containing the caudate was glued to the chuck of a vibratome. Coronal sections (400 μm) were made through the caudate and transferred into a mesh slice saver immersed in aCSF bubbled with 95% O_2_/5% CO_2_, at room temperature (21±1 °C). The slices were equilibrated in the slice saver to allow recovery from trauma associated with slicing (Toner and Stamford, 1996). We took multiple slices from each mouse (typically 2), but each slice was used in a different experiment such that in one experimental group each slice was from a different mouse.

Following equilibration, slices were transferred to the slice chamber (33.0±0.5 °C) and continuously superfused with aCSF (250 ml/h) for 45 min. The perfusion medium was then switched to OGD aCSF for 0–40 min depending on the experiment (see later) after which slices were superfused for a further 60 min in aCSF and then exposed to a second OGD event.

The composition of aCSF was *Maintenance aCSF* (mM): NaCl (126.0), KCl (2.0), KH_2_PO_4_ (1.4), MgSO_4_ (2.0), NaHCO_3_ (26.0), CaCl_2_ (2.4), (+)-glucose (10.0), bubbled for at least 60 min with 95% O_2_/5% CO_2_. *OGD aCSF* (mM): NaCl (1 2 6), KCl (2.0), KH_2_PO_4_ (1.4), MgSO_4_ (2.0), NaHCO_3_ (26.0), CaCl_2_ (2.4), (+)-glucose (2.0), bubbled for at least 60 min with 95% N_2_/5% CO_2_.

### 2.3 Fast cyclic voltammetry (FCV)

Extracellular dopamine concentrations in the dorso-lateral caudate nucleus were measured by FCV at carbon fibre microelectrodes. Carbon electrodes were made by inserting an 8 μm diameter carbon fibre into a 10 cm length borosilicate glass capillary (o.d., 2.0 mm; i.d., 1.16 mm: Harvard Apparatus, UK), which was pulled using an PE21 electrode puller (Narishige, Japan), such that the carbon fibre protruded from the pulled tip. The carbon fibre was then cut to a length of 75 μm.

**Fig. 1:**
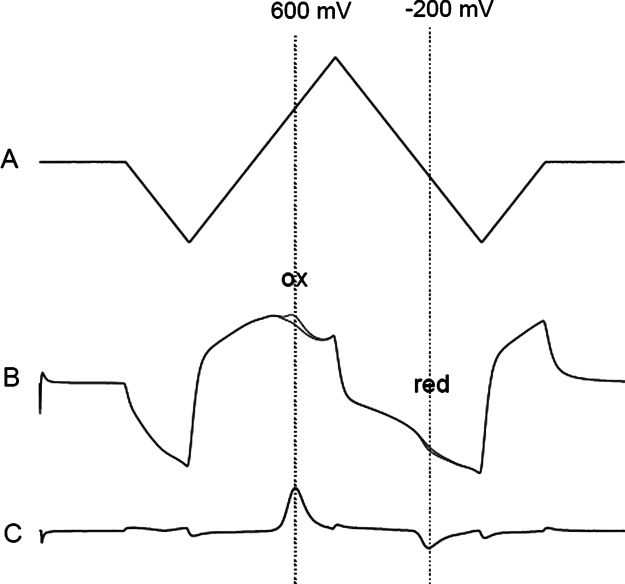
Voltammetry input voltage waveform, current at carbon electrode and subtracted voltammogram showing dopamine oxidation and reduction peaks. (A) Input voltage waveform to carbon electrode. The voltage scan goes from 0 to −1 to +1.4 to −1 and back to 0 V at 480 V/s. The whole scan takes 20 ms. (B) The current at the carbon electrode after applying the input voltage in aCSF and in the presence of 10 μM dopamine. The two scans are superimposed except for a small increase at around 600 mV (where dopamine oxidises giving off two electrons) and at −200 mV where dopamine is reduced. (C) The voltammogram is derived from B and obtained by subtracting the current at the electrode in aCSF from the current at the electrode in the presence of dopamine, leaving only the Faradaic current from dopamine oxidation and reduction. Note the oxidation peak at 600 mV and the reduction peak at −200 mV, indicative of dopamine in the caudate.

A stainless steel auxiliary electrode and a Ag/AgCl reference electrode were placed in the slice chamber remote from the slice. Voltammetric scans (−1.0 to +1.4 V vs Ag/AgCl, 480 V/s) were applied at 1 Hz using a Millar voltammeter (PD Systems, UK). Under these conditions dopamine oxidised at +600 mV and reduced at −200 mV (Fig. 1). Voltammetric scans were saved using Clampex 9.0 (Molecular Devices, USA). Following each experiment, the electrode was calibrated in dopamine (10 μM), and measurements made during the experiments were converted to dopamine concentrations.

**Fig. 2:**
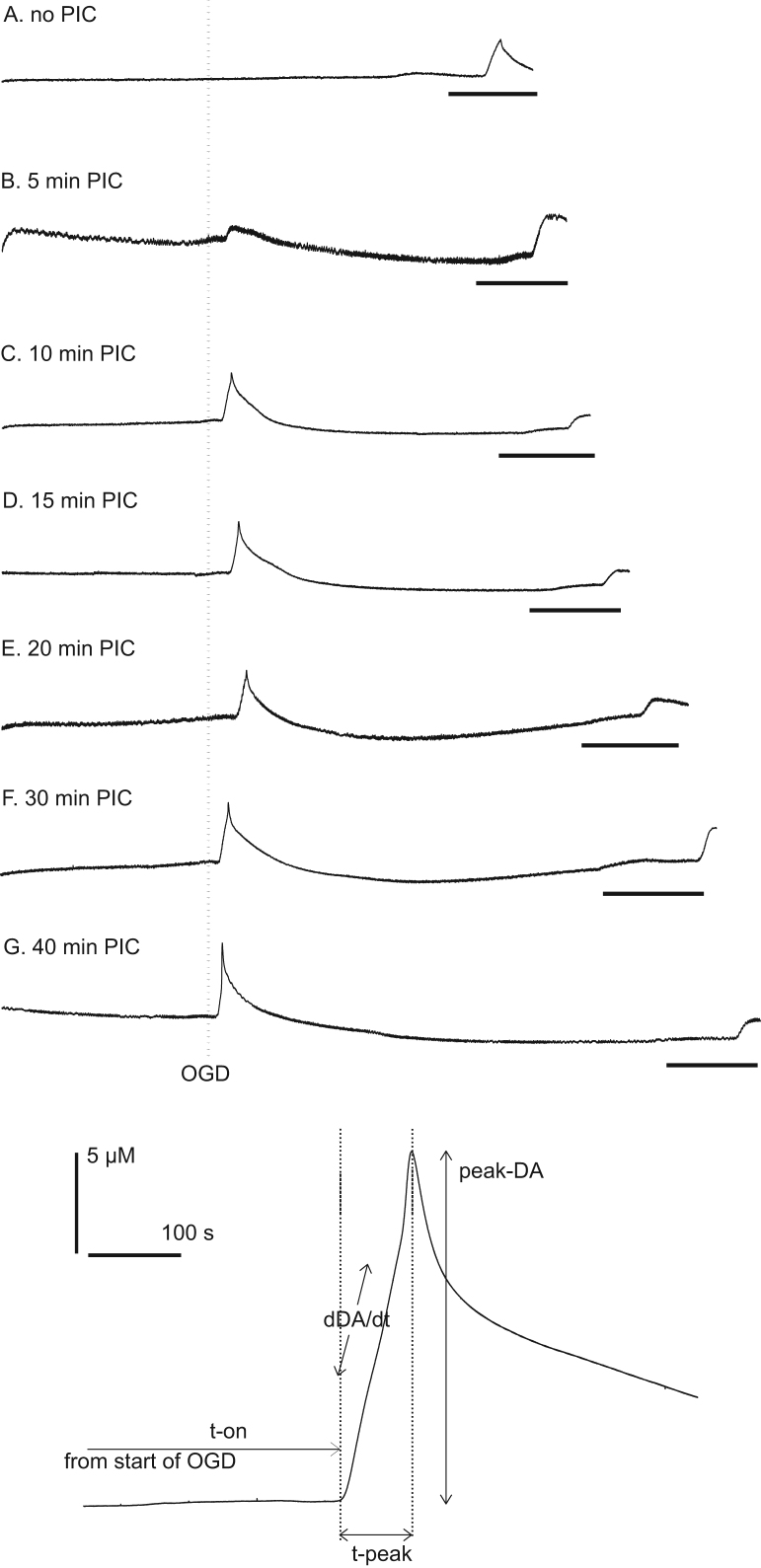
Raw data and variables measured during conditioning experiments. Top panel shows typical experiments for 5–40 min PIC followed 60 min later by a second OGD event (black bar, 20 min). The vertical dotted line shows the start of the first OGD event (i.e. the PIC event). The caudate brain slice is superfused with oxygenated aCSF for 45 min and then subjected to 0–40 min OGD. After about 5 min we see a massive and fast increase in dopamine of approximately 15 μM (C–G). Panel A shows 125 min of data, with subsequent panels showing slightly longer traces, depending on the length of PIC, up to 165 min for panel G. The lower panel shows the dopamine efflux event in more detail and the parameters we measure; T-on is the time from the start of OGD until dopamine efflux; T-peak is the time from the start of dopamine efflux until peak dopamine efflux; peak-DA is the maximum dopamine concentration measured; *δ*DA/*δt* is the rate of change of dopamine efflux (nM/s).

After the slice was placed in the slice chamber, the electrode tip was positioned approximately 100 μm below the slice surface in the dorso-lateral caudate, using a micromanipulator. Recording started immediately as this allowed us to monitor the stability of the slice as on some occasions (e.g. poor slice health) the slice can spontaneously release large amounts of dopamine (Davidson et al., 2011a). Perfusion with OGD aCSF typically evoked a large increase in dopamine from the slice (Fig. 2) and four parameters of dopamine release were measured (1) time to onset of dopamine release from the initiation of OGD (T-on); (2) time taken to reach maximum dopamine release after the onset of release (T-peak); (3) maximum extracellular dopamine concentration (peak-dopamine); and (4) mean rate of dopamine release (*δ*DA/*δt*) as previously described (Toner and Stamford, 1997a, b, Davidson et al., 2011a, b).

### 2.4 Pre-ischeamic conditioning protocol.

#### 2.4.1 Protocol 1: effect of length of PIC event on evoked tolerance

**Fig. 3:**
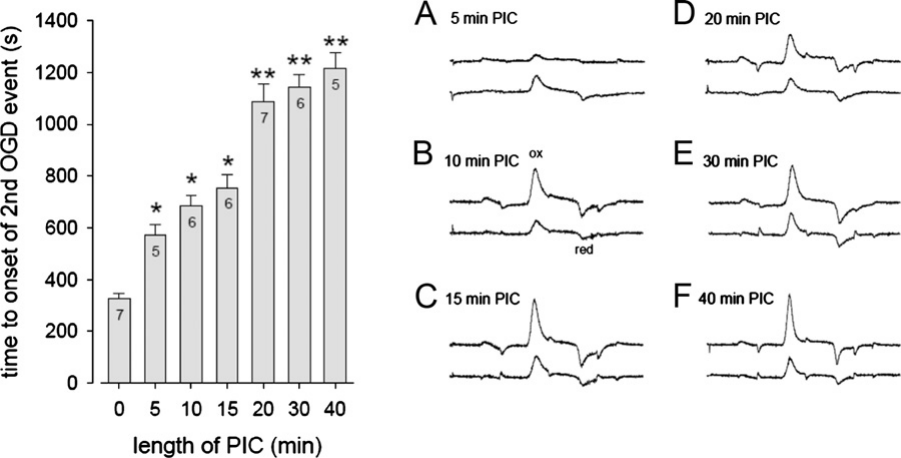
Time to onset of dopamine efflux and voltammograms from the conditioning and final OGD events. Left panel: As the length of the conditioning event increased from 0 to 40 min OGD there was a corresponding increase in T-on of OGD-evoked dopamine efflux on the second OGD event 60 min later (Fig. 2 upper panel for raw data). ^⁎^*P*<0.05 vs 0 min; ^⁎⁎^*P*<0.05 vs 0, 5, 10 and 15 min. *N*=5–7 (given in individual columns). Right panel A–F shows the oxidation and reduction peaks for the first OGD event in the top trace, while the lower traces show the peaks for the second OGD event. Note that in all cases, except 5 min PIC, the upper trace dopamine oxidation peak is larger than the lower trace i.e. less dopamine is released on the second OGD event. The upper traces are offset to aid clarity. The 20 ms of data is shown in each trace which corresponds to the voltage input waveform shown in Fig. 1A. Dopamine oxidation peaks are at approximately 600 mV and reduction peaks are at −200 mV. These data show that we are measuring dopamine both at the first and second OGD events. The initial OGD-evoked oxidation peaks correspond to approximately 20 μM (except panel A which is lower).

After 45 min equilibration a slice was exposed to 0, 5, 10, 15, 20, 30 or 40 min OGD. Thereafter the slice was exposed to 60 min of oxygenated aCSF and then exposed to a second OGD event of at least 20 min duration. Thus we could examine the effect PIC (0–40 min) on the second OGD event 60 min later (Figs. 2 and 3).

#### 2.4.2 Protocol 2: effect of NMDA- or adenosine-A_1_ receptor antagonists on PIC

We measured the effect of the adenosine-A_1_ receptor antagonist 8-cyclopentyl-1,3-dipropylxanthine (DPCPX; 5 μM) and of the NMDA receptor antagonist, MK-801 (10 μM) on the tolerance evoked by 10 min PIC. The slice was superfused with aCSF containing either DPCPX or MK-801 for 45 min. The superfusate was then changed to OGD aCSF containing the respective drug, for 10 min, after which the superfusion aCSF was switched to drug-free aCSF for 60 min, then to drug-free OGD for at least 20 min. We used a second OGD event of 20 min or more (vs 10 min for the conditioning event) because in preliminary experiments the OGD-evoked dopamine efflux was found to be delayed to such an extent that on some occasions 10 min OGD is not long enough to evoke dopamine efflux.

On completion of these protocols some slices were removed from the slice chamber and prepared for TTC staining to assess mitochondrial function, or for HPLC analysis of tissue content of dopamine, as described below.

### 2.5 Triphenyltetrazolium chloride (TTC) staining

After OGD superfusion, slices were incubated in aCSF at room temperature (21±1 °C) for 30 min, then transferred into sterile cell culture wells and stained with TTC (2%w/v in 0.9% NaCl) for 30 min in the absence of light. The slices were then fixed with 4% paraformaldehyde solution, photographed and the percentage of the caudate area stained was assessed using Image software (Beta 4.0.2, Scion Corporation, USA).

### 2.6 High performance liquid chromotography (HPLC)

On termination of FCV experiments, slices for HPLC analysis were removed from the chamber and the caudate dissected out on ice, placed in a microcentrifuge tube and weighed. Perchloric acid (0.2 N, 500 μl) was added to the tube and the tissue was homogenised for 20 s (Tissue-tearor; Biospec Products). The homogenate was centrifuged (6000 rpm, 30 min, 21 °C), and the supernatant taken for HPLC analysis. A 15 μl aliquot of sample was injected automatically and separation was achieved on a C18 Luna reverse phase column (100×1.0 mm; 5 μm; Phenomenex), with a mobile phase comprising 75.0 mM NaH_2_PO_4_, 1.2 mM octane sulphonic acid, 1.0 mM EDTA and 10% methanol, pH 3.7. The mobile phase was delivered at 110 μl/min, using a Jasco high pressure pump. Electrochemical detection was at a glassy carbon working electrode, set at 700 mV relative to Ag/AgCl, using an Antec Intro electrochemical detector incorporating a VT-03 low volume flow cell (Antec Instruments). The concentration of dopamine was measured relative to standard solutions (100 nM), using ChromPerfect data analysis software (Justice Laboratory). Each sample was run in duplicate, and the mean tissue content of dopamine was calculated and normalised to the wet weight of tissue in nM/mg.

### 2.7 Drugs and chemicals

All chemicals were purchased from Sigma Aldrich. DPCPX was initially dissolved in DSMO to make a stock solution of 10 mM, and then diluted in aCSF to give a working concentration of 5 μM, a concentration that has previously been shown to attenuate anoxia-evoked PIC in hippocampal brain slice preparations (Perez-Pinzon et al., 1996). MK-801 was initially dissolved in deionised water to a stock solution of 10 mM and then diluted in aCSF to give a working concentration of 10 μM. We found that control or PIC experiments with DMSO control (DPCPX vehicle controls) were no different from these experiments without DMSO (MK-801 vehicle control) and so all vehicle control experiments were combined.

### 2.8 Statistics

Where the effects of one condition were to be compared with another, a two-tailed, unpaired student t-test was done. When there were more than two conditions with one variable, a one-way analysis of variance (ANOVA) was done with post hoc analysis with Tukey's post test for multiple comparisons. A two-way ANOVA was done when two independent factors were present. A probability of *P*<0.05 was considered significant. SigmaStat software was used for all statistical analysis. Values are means±SEM.

## 3 Results

Consistent OGD-evoked dopamine release occurred 350±82 s after the onset of OGD, taking 148±17 s to achieve peak-dopamine efflux of 20.3±4.0 μM. The rate of change of dopamine efflux was 73±19 nM/s (Fig. 3 and Table 1).

### 3.1 Effect of pre-ischeamic conditioning length on the second OGD event.

Single PIC events of 5–40 min evoked a significant delay in T-on of OGD-evoked dopamine release 60 min later (*F*(6,41)=46.88, *P*<0.05); Fig. 3). The 5 min PIC event also lengthened T-peak from about 148 to 345 s (*F*(6, 41)=3.867, *P*<0.05) but the other PIC events (10–40 min) had no effect on T-peak. All PIC events (5–40 min) significantly reduced the OGD-evoked peak-dopamine, from 20.3±4.0 μM in control slices to 3.6±1.1 μM 60 min after 40 min PIC, *F*(6, 41)=7.58, *P*<0.05. All PIC events (5–40 min) significantly reduced *δ*DA/*δt*, from 73 nM/s in control slices to 19 nM/s 60 min after 40 min PIC (*F*(6, 41)=4.39, *P*<0.05; Table 1).

### 3.2 Effect of post-PIC incubation on caudate tissue dopamine content

**Fig. 4:**
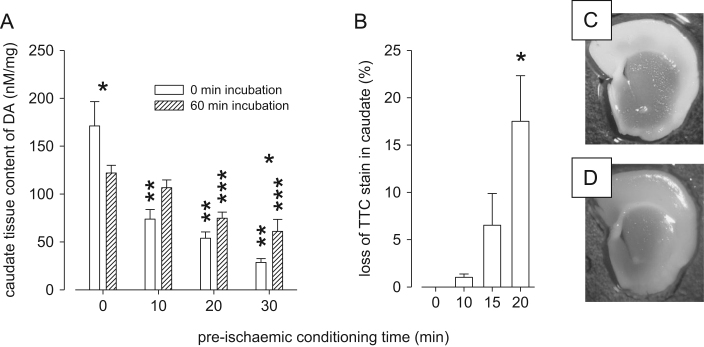
Effect of different conditioning periods and reperfusion on caudate dopamine content and TTC staining. (A) *HPLC data*: Caudate slices were superfused with aCSF for 45 min then exposed to 0–30 min OGD. Immediately after OGD (0 min incubations in figure legend) the caudate was harvested while in other experiments the slice was allowed to recover for 60 min after OGD (60 min incubation in the figure legend) after which the caudate was harvested. When the caudate was examined with 0 min incubation after OGD we found that 10, 20 and 30 min of OGD caused a reduction in dopamine content (^⁎⁎^*P*<0.05 vs 0 min PIC), however, in the 60 min incubation slices only the 20 and 30 min OGD slices were different from controls (^⁎⁎⁎^*P*<0.05 vs control). In control experiments with 0 min OGD dopamine content fell by about 30% after a further 60 min incubation (^⁎^*P*<0.05). When comparing the caudate immediately after 30 min OGD with that after a further 60 min incubation it was found that the dopamine content recovered, although not quite to control levels (^⁎^*P*<0.05 0 vs 60 min incubation after 30 min PIC). *N*=4–8. (B) *TTC data*: In similar experiments we examined TTC staining in caudate slices that had been exposed to 0–20 min OGD and then 60 min incubation. Only 20 min OGD caused a loss of TTC staining 60 min later (^⁎^*P*<0.05 vs 0 min OGD). Thus 10 and 15 min OGD do not appear to cause permanent damage to the slice whereas 20 min OGD does appear to cause mitochondrial dysfunction for at least 60 min after the end of OGD. *N*=4–6. Right panel: (C) control caudate slice stained with TTC and (D) a slice stained 60 min after a 20 min OGD event showing a loss of staining.

It is possible that less dopamine is evoked on the second OGD event because there is less dopamine in the dopamine terminals either because (1) much was released on the first OGD event or (2) the slices' ability to synthesise dopamine was now compromised. We therefore examined caudate dopamine tissue content immediately after the first OGD event and, in different slices, after 60 min incubation following OGD. The data show that dopamine tissue content is reduced after OGD, but that it recovers to near control levels after 60 min reperfusion (Fig. 4A).

There was a main effect of PIC time (*F*(3, 33)=29.95, *P*<0.05) and a PIC time X incubation interaction (*F*(3,33)=5.79, *P*<0.05). Tukey's test revealed that, immediately after the PIC event, all three PIC treatments (10, 20 and 30 min) had reduced the dopamine content vs no PIC (all, *P*<0.05). However, after 60 min reperfusion dopamine content partially recovered in that now only the 20 and 30 min PIC was different from controls (both *P*<0.05, Fig. 4A). Within the control group (0 min PIC) dopamine content fell during the 60 min reperfusion (*P*<0.05) while in the 30 min PIC group dopamine content increased over the 60 min reperfusion (*P*<0.05) and there was a tendency for dopamine content to increase after reperfusion in the 10 min PIC group (*P*=0.057). These data suggest that even after 30 min of OGD the slice can recover its dopamine synthesising capacity.

### 3.3 Effect of post-PIC incubation on TTC staining in the caudate

In order to examine whether the first OGD event was neurotoxic we undertook TTC staining to assess mitochondrial activity. Slices were exposed to OGD for 0, 10, 15 or 20 min then reperfused for 60 min. There was a significant difference in loss of TTC staining (*F*(3, 15)=6.67, *P*<0.05) and Tukey's test revealed that there was a loss of TTC staining after 20 min OGD vs both 0 and 10 min OGD (*P*<0.05, Fig. 4B). Taken together with the FCV and HPLC data, 10 min PIC evoked tolerance allowed recovery of dopamine content and did not cause a loss of TTC staining, thus we used this conditioning time in subsequent experiments.

### 3.4 Does MK-801 block conditioning?

**Fig. 5:**
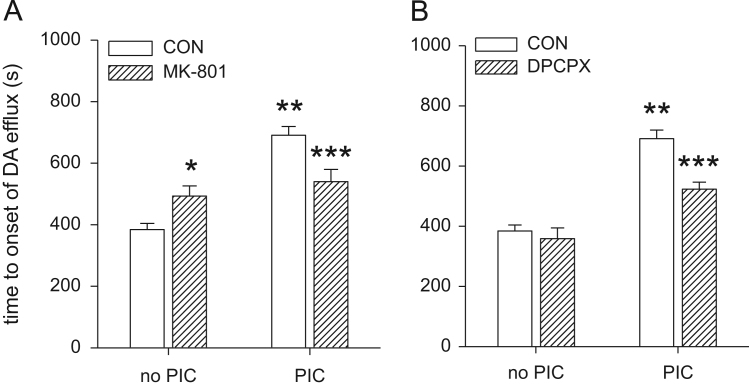
Effect of MK-801 or DPCPX on OGD and conditioning. Caudate slices were superfused in either vehicle, MK-801 or DPCPX for 45 min then exposed to a conditioning event of either 0 or 10 min OGD, during which the vehicle, MK-801 or DPCPX was still present. The slices were then superfused with normal aCSF for a further 60 min and exposed to a second OGD. The T-on of the second OGD evoked dopamine efflux event are given above (see Table 2 for other parameters). (A) 10 min PIC with OGD-evoked tolerance to a second OGD event 60 min later (^⁎⁎^*P*<0.05). MK-801 (10 μM) increased T-on (^⁎^*P*<0.05). In the presence of MK-801 the tolerance evoked by 10 min PIC was attenuated (^⁎⁎⁎^*P*<0.05). (B) As in panel A, 10 min PIC evoked tolerance to a second OGD event 60 min later (^⁎⁎^*P*<0.05). DPCPX had no effect on OGD but attenuated the increase in T-on evoked by PIC (^⁎⁎⁎^*P*<0.05). *N*=6–12 slices for each group.

We superfused the caudate slice with MK-801 for 45 min prior to 10 min OGD conditioning (with MK-801 present during PIC). We then subjected the slice to a second OGD event 60 min later and measured the dopamine efflux parameters on the second OGD-evoked dopamine release event. PIC increased T-on after OGD (*F*(1, 38)=37.43, *P*<0.05). There was no main effect of MK-801 (*F*(1, 38)=0.01, *P*=0.93) but there was a PIC × MK-801 interaction (*F*(1, 38)=16.12, *P*<0.05). Post hoc analysis revealed that PIC increased T-on in the absence of MK-801 (*P*<0.05), but there was no PIC-induced increase in T-on in the presence of MK-801 (*P*=0.18). MK-801 also increased T-on in the absence of PIC (*P*<0.05). Thus, MK-801 increases T-on in caudate slices that have not been conditioned, but attenuates the increase in T-on after PIC (Fig. 5A, Table 2).

MK-801 had no effect on T-peak (*F*(1, 38)=2.48, *P*=0.13), nor did PIC (*F*(1, 38)=0.5, *P*=0.49) and there was no PIC × MK-801 interaction (*F*(1, 38)=0.93, *P*=0.34). PIC did reduce peak-dopamine (*F*(1, 38)=30.6, *P*<0.05) but MK-801 had no effect on peak-dopamine (*F*(1, 36)=0.069, *P*=0.89) and there was no PIC × MK-801 interaction (*F*(1, 36)=0.14, *P*=0.71). PIC decreased *δ*DA/*δt* (*F*(1, 38)=31.8, *P*<0.05) as did MK-801 (*F*(1, 38)=16.5, *P*<0.05) and there was a PIC X MK-801 interaction (*F*(1, 38)=9.7, *P*<0.05). Thus MK-801 slowed down the rate of dopamine efflux in slices which had not been exposed to PIC, but had no effect in slices after PIC (Table 2). Taken together, in the absence of PIC, MK-801 slows down the onset of OGD-evoked dopamine efflux and slows down the rate of dopamine efflux.

### 3.5 Does DPCPX block conditioning?

We superfused the caudate slice with DPCPX for 45 min prior to 10 min PIC (with DPCPX present during PIC). We then subjected the slice to a second OGD event 60 min later and measured the dopamine efflux parameters on the second OGD-evoked dopamine release event. ANOVA revealed a PIC X DPCPX interaction (*F*(1, 34)=7.69, *P*<0.05). Thus PIC increased T-on (*p*<0.05) and DPCPX reduced the PIC-induced increase in T-on (*P*<0.05). In the absence of PIC, DPCPX had no effect on T-on (Fig. 5B). Neither PIC nor DPCPX had any effect on T-peak (both *P*>0.3) and there was no effect of DPCPX on *δ*DA/*δt* (*P*>0.7) or on peak-dopamine (*P*>0.5; Fig. 5B, Table 2).

### 3.6 Effect of MK-801 and DPCPX on caudate TTC staining.

**Fig. 6:**
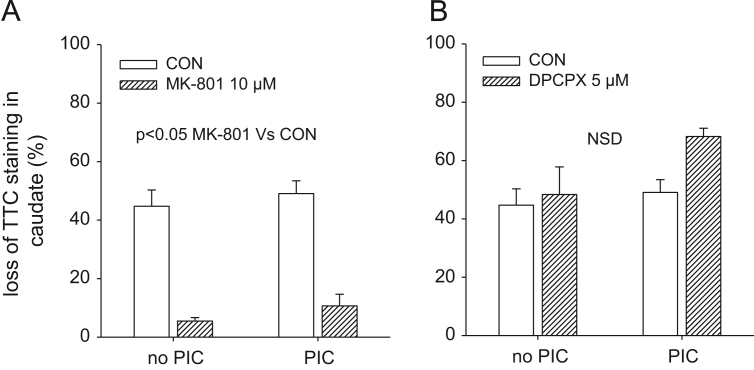
Effect of MK-801 and DPCPX on TTC staining in the caudate with and without PIC. Caudate slices were first superfused for 45 min with vehicle, MK-801 (10 μM) or DPCPX (5 μM) then exposed to either 0 or 10 min PIC, 60 min later they were exposed to 20 min OGD and the tissue was stained with TTC. (A) Immediately after the final OGD event there was an approximate 40% loss in TTC staining, MK-801 attenuated this loss. PIC had no effect on the loss of TTC staining. (B) DPCPX had no effect on TTC staining. *N*=4–8, PIC=pre-ischaemic conditioning.

There was no significant effect of PIC on caudate TTC staining immediately after the final OGD event (*F*(1, 23)=0.88, *P*=0.36), however, MK-801 reduced the OGD-evoked loss of TTC staining (*F*(1, 23)=89.5, *P*<0.05). There was no interaction between PIC and MK-801 (*F*(1, 23)=0.01, *P*=0.91). Thus MK-801 attenuated the loss of TTC staining regardless of whether the slice had been exposed to PIC or not (Fig. 6A). There was no significant effect of DPCPX on the loss of TTC staining in the caudate immediately after the final OGD event (*F*(1, 21)=2.55, *P*=0.13) and no interaction between PIC and DPCPX (*F*(1, 21)=1.18, *P*=0.29; Fig. 6B).

## 4 Discussion

We have found that longer conditioning events evoke greater tolerance to a second OGD event, but possibly at the cost of some neurotoxicity as determined by TTC staining. We have also shown that even a 30 min PIC event is not “lethal” as the slice was still able to synthesise dopamine for up to 60 min after PIC. Our data suggest that, under these conditions, 10 or 15 min PIC might afford the best balance between evoking tolerance and not causing irrevocable toxicity. We then examined receptor mechanisms underlying this conditioning phenomenon. We first examined the NMDA receptor antagonist MK-801 both in the absence and presence of 10 min PIC. In the absence of PIC, MK-801 increased T-on, but decreased T-on 60 min after 10 min PIC. Our findings are in agreement with previous work that has demonstrated the neuroprotective profile of NMDA receptor antagonists when examining the effects on OGD-evoked dopamine release, although previous work has not examined PIC. NMDA receptor antagonists CGS 19755, MK-801 (Toner and Stamford, 1997b) and ketamine (Mathews et al., 2001) have been shown to increase T-on. MK-801 also increased T-peak, as did ketamine (Toner and Stamford, 1997b, Mathews et al., 2001). The NMDA receptor has an inhibitory Mg^2+^ binding site and increasing [Mg^2+^] from 1.3 to 3.7 mM increased T-peak and decreased *δ*DA/*δt* (Toner and Stamford, 1997b). Here, we also show that MK-801 attenuates PIC. These data reveal a complex situation in the rodent caudate where NMDA receptor activation may have both toxic and neuroprotective consequences, presumably dependent upon the degree of NMDA receptor activation. We also found MK-801 to decrease the loss of TTC staining associated with OGD, consistent with MK-801 having a neuroprotective profile. Adenosine is another transmitter found in high levels in the caudate and has often been implicated in conditioning effects in the hippocampus and cortex (Williams-Karnesky and Stenzel-Poore, 2009). We examined the adenosine A_1_ receptor antagonist DPCPX, which had no effect on T-on or TTC staining during OGD-only but reduced the effect of PIC on T-on. This suggests that, in the caudate, the adenosine A_1_ receptor, unlike the NMDA receptor, appears to be only involved in PIC and not neuroprotection per se.

A possible explanation for our finding that PIC delays T-on could be through OGD-evoked adenosine A_1_ receptor activation. Activation of this receptor is proposed to lead to activation of protein kinase C, and opening of mitochondrial *K*_ATP_ channels. Hyperpolarisation of the neuron and down-regulation of the neuronal metabolic and energy dependent mechanisms have been thought to ensue from opening the neuronal *K*_ATP_ channels (Nakagawa et al., 2002). Furthermore, opening of the mitochondrial *K*_ATP_ channels may depolarise the mitochondrial membrane, leading to an increase in the electron transport chain activity, and consequently an increase in ATP production (Inoue et al., 1991; Perez-Pinzon, 2007). NMDA receptor activation may activate similar downstream processes (Dirnagl et al., 2003). As a result of the increased energy availability during a secondary ischeamic event, either through an increase in ATP synthesis or a decrease in ATP usage, energy depletion may be delayed. A delay in the reduction in ATP during OGD would postpone the subsequent steps in the ischeamic cascade, including anoxic depolarisation and as a result, caudate dopamine efflux.

In addition to increased T-on, other parameters can be associated with putative neuroprotection including T-peak, peak-dopamine and *δ*DA/*δt* (Toner and Stamford, 1996). Five-minutes PIC not only delayed T-on but also delayed the T-peak. A delay in T-peak could be considered neuroprotective by providing more time for neuronal uptake of released dopamine or diffusion of dopamine away from the ischeamic site, decreasing peak dopamine concentrations. This could potentially prevent dopamine reaching neurotoxic levels (Lieb et al., 1995). However, dopamine reuptake mechanisms have been shown to be compromised during ischaemia (Kondoh et al., 1995) and it is questionable how much is taken back up through the transporter during or after OGD. All PIC events (5–40 min) reduced peak-dopamine on a subsequent OGD event 60 min later and an obvious explanation would be reduced dopamine content in the slice after the initial PIC-evoked dopamine efflux. However, HPLC analysis of dopamine tissue content after 60 min post-OGD incubation showed that 10 min of ischaemia did not significantly reduce dopamine content. Further, dopamine content increased during the 60 min reperfusion after a 30 min OGD event. Thus the decrease in peak dopamine found during OGD 60 min after PIC would appear to be due to tolerance rather than a reduction in caudate dopamine tissue content. This could be explained by the release of adenosine during ischaemia which has been shown to hyperpolarise presynaptic membranes, inhibiting further neurotransmitter release (de Mendonça et al., 2000). Furthermore, PIC lowers calcium influx during a subsequent ischaemia leading to a putative reduction in neurotransmitter release (Shimazaki et al., 1998).

We have found MK-801 to attenuate PIC in the caudate. Blockade of PIC by NMDA receptor antagonists has been achieved in vivo in the hippocampus (Chen et al., 2008; Bond et al., 1999) and in vitro (Grabb and Choi, 1999). Furthermore, preconditioning with NMDA improved synaptic recovery in vitro (Youssef et al., 2006) and provided reductions in infarct area after microinjections in the prefrontal cortex in vivo (Saleh et al., 2009). The present study showed that PIC-evoked neuroprotection was attenuated in the presence of MK-801, suggesting that NMDA receptor activation may play a role in rapid (within minutes to hours) PIC, in the caudate. We also found that the application of DPCPX prior to and during PIC attenuated the protective effect of PIC. The blockade of adenosine A_1_ receptor with DPCPX has been shown to attenuate PIC in a delayed (Reshef et al., 2000) and rapid (Nakamura et al., 2002) latency period. Furthermore, mechanisms thought to be downstream of adenosine A_1_ receptor activation; PKC and K_ATP_ channel activation, have been elucidated in a variety of models (Raval et al., 2007). Since increased synthesis and reduced utilisation of ATP could result from adenosine A_1_ receptor activation, the attenuation of this would result in reduced T-on, which was seen after PIC in the presence of DPCPX. However, as the delay in T-on was not fully abolished this suggests either (1) adenosine A_1_ receptors were not sufficiently blocked or (2) the delay in T-on found is mediated through additional mechanisms e.g. the NMDA receptor. Even though the delay in T-on was not fully attenuated the results suggest that during PIC, activation of adenosine A_1_ receptors is involved in the cellular pathway responsible for the tolerance to an OGD insult in the caudate.

There is an apparent mismatch between our voltammetry and TTC data in that PIC evokes tolerance to a second OGD event but no apparent tolerance to toxicity, as determined by TTC staining (Fig. 6). Thus although PIC caused a delay in T-on for the second OGD event, it had no effect on TTC staining immediately after the second OGD event. Clearly the caudate tissue is showing tolerance and this questions what the TTC stain is telling us. TTC turns red when the mitochondrial dehydrogenase enzymes react with tetrazolium salts to form a formazan pigment. Thus, the lack of TTC staining could simply reflect tissue that is compromised rather than tissue that is dead or dying. The fact that we found a 45% loss of TTC staining immediately after the OGD event (Fig. 6) but only a 15% loss of TTC staining after 60 min reperfusion (Fig. 4) suggests that the tissue shows some recovery after this OGD event. Others have found similar recovery in TTC staining on reperfusion (Mathews et al., 2000). Thus, although PIC does not evoked tolerance to the loss of TTC staining, we still believe that tolerance to OGD has occurred, as shown by the increase in T-on.

## 5 Conclusions

Attenuating or slowing ischaemia-evoked dopamine efflux may limit neuronal damage after stroke. We have shown that brief exposure to OGD in vitro elicits rapid adaptive mechanisms that delay the onset of dopamine efflux during a secondary OGD episode. We propose that increases in T-on and T-peak are indicative of neuroprotection as the brain is more tolerant to subsequent OGD. Our main finding was that the tolerance achieved with 10 min conditioning was attenuated in the caudate by the application of either the adenosine A_1_ receptor antagonist, DPCPX or the glutamate-NMDA receptor antagonist, MK-801. Given that adenosine A_1_ and NMDA receptors are likely to be involved in PIC; these could be future targets to induce neuroprotection pharmacologically. These data, involving OGD-evoked dopamine efflux in the caudate, add to the wealth of data on PIC from the hippocampus and cortex and suggest receptor mechanisms that may be involved in PIC throughout the brain.

## Figures and Tables

**Table 1:** Effect of increasing the conditioning period on dopamine efflux after a second ODG event 60 min later.

DA efflux parameter	Pre-ischaemic conditioning time (min)						
	0 (*n*=7)	5 (*n*=5)	10 (*n*=6)	15 (*n*=6)	20 (*n*=7)	30 (*n*=6)	40 (*n*=5)
T-peak (s)	148±17	345±55^⁎^	194±24	167±20	242±36	239±26	258±47
Peak-DA (μM)	20.3±4.0^a^	8.1±1.1	5.0±0.8	8.3±2.1	5.1±1.4	5.0±1.3	3.6±1.1
*δ*DA/*δt* (nM/s)	73±19^b^	26±7	29±4	46±8	21±6	22±6	19±9

In addition to the effects of various conditioning times on T-on (Fig. 3), other dopamine efflux parameters are shown in this table. ^⁎^*P*<0.05 vs 0 and 15 min PIC.

a *P*<0.05 vs all other groups.

b *P*<0.05 vs all other groups except 15 min PIC. T-peak=time from the start of dopamine efflux to peak efflux; peak-DA=peak dopamine efflux; *δ*DA/*δt*=rate of change of dopamine efflux.

**Table 2:** Effect of MK-801 and DPCPX on OGD-evoked dopamine efflux and conditioning effects. The dopamine efflux parameters of the second OGD-evoked dopamine efflux event are given above (see Fig 5 for T-on).

Efflux parameters	No conditioning		10 min PIC	
	No drug	MK-801 (10 μM)	No drug	MK-801 (10 μM)
***MK-801 analysis***				
T-peak (s)	176±17	128±23	171±17	159±18
Peak-DA (μM)^⁎^	15.5±1.5	18.0±1.9	5.7±1.6	9.4±1.7
*δ*DA/*δt* (nM/s)^⁎⁎⁎^	90±17	241±26^a^	37±19^b^	57±21^c^
				
***DPCPX analysis***				
	No drug	DPCPX (5 μM)	No drug	DPCPX (5 μM)

T-peak (s)	176±20	146±26	171±20	195±23
Peak-DA (μM)^⁎^	15.5±1.5	11.4±1.8	5.7±1.6	7.6±1.8
*δ*DA/*δt* (nM/s)^⁎^	90±17	95±20	37±19	47±18

^⁎^*P*<0.05 main effect of PIC; ^⁎⁎⁎^*P*<0.05 main effect of PIC and MK-801 and a PIC X MK-801 interaction, T-peak=time from the start of dopamine efflux to peak efflux; peak-DA=peak dopamine efflux; *δ*DA/*δt*=rate of change of dopamine efflux. OGD=oxygen and glucose deprivation; PIC=pre-ischaemic conditioning. *N*=6–12 (see Fig 5).

a *P*<0.05 no drug vs MK-801 with no conditioning i.e. MK-801 slows down the rate of OGD-evoked dopamine efflux.

b *P*<0.05 PIC vs no PIC (in the absence of MK-801) i.e. PIC reduces the rate of dopamine efflux.

c *P*<0.05 PIC vs no PIC (in the presence of MK-801) i.e. PIC still slowed down the rate of dopamine efflux even with MK-801 present.
